# Prevalence and Therapy Rates for Stuttering, Cluttering, and Developmental Disorders of Speech and Language: Evaluation of German Health Insurance Data

**DOI:** 10.3389/fnhum.2021.645292

**Published:** 2021-04-12

**Authors:** Martin Sommer, Andrea Waltersbacher, Andreas Schlotmann, Helmut Schröder, Adam Strzelczyk

**Affiliations:** ^1^Bundesvereinigung Stottern & Selbsthilfe e.V., German Stuttering Association, Cologne, Germany; ^2^Department of Clinical Neurophysiology, University Medical Center Göttingen, Göttingen, Germany; ^3^Department of Neurology, University Medical Center Göttingen, Göttingen, Germany; ^4^Wissenschaftliches Institut der AOK (WIdO), AOK Research Institute, Berlin, Germany; ^5^Epilepsy Center Frankfurt Rhine-Main, Department of Neurology and Neurosurgery, Goethe University Frankfurt, Frankfurt, Germany

**Keywords:** stuttering, cluttering, morbidity, epidemiology, secondary data analysis

## Abstract

**Purpose:**

To evaluate the prevalence and treatment patterns of speech and language disorders in Germany.

**Methods:**

A retrospective analysis of data collected from 32% of the German population, insured by the statutory German health insurance (AOK, Local Health Care Funds). We used The International Statistical Classification of Diseases and Related Health Problems, 10th revision, German Modification (ICD-10 GM) codes for stuttering (F98.5), cluttering (F98.6), and developmental disorders of speech and language (F80) to identify prevalent and newly diagnosed cases each year. Prescription and speech therapy reimbursement data were used to evaluate treatment patterns.

**Results:**

In 2017, 27,977 patients of all ages were diagnosed with stuttering (21,045 males, 75% and 6,932 females, 25%). Stuttering prevalence peaks at age 5 years (boys, 0.89% and girls, 0.40%). Cluttering was diagnosed in 1,800 patients of all ages (1,287 males, 71.5% and 513 females, 28.5%). Developmental disorders of speech and language were identified in 555,774 AOK-insurants (61.2% males and 38.8% females). Treatment data indicate a substantial proportion newly diagnosed stuttering individuals receive treatment (up to 45% of 6-year-old patients), with slightly fewer than 20 sessions per year, on average. We confirmed a previous study showing increased rates of atopic disorders and neurological and psychiatric comorbidities in individuals with stuttering, cluttering, and developmental disorders of speech and language.

**Conclusion:**

This is the first nationwide study using health insurance data to analyze the prevalence and newly diagnosed cases of a speech and language disorder. Prevalence and gender ratio data were consistent with the international literature. The crude prevalence of developmental disorders of speech and language increased from 2015 to 2018, whereas the crude prevalence for stuttering remained stable. For cluttering, the numbers were too low to draw reliable conclusions. Proportional treatment allocation for stuttering peaked at 6 years of age, which is the school entrance year, and is later than the prevalence peak of stuttering.

## Introduction

Stuttering is a speech fluency disorder that presents with repetitions, prolongations of sounds and syllables, and speech blocks. Verbal or situational avoidance behavior and involuntary movements may develop over time in patients diagnosed with stuttering (Mulligan et al., 2001; American Psychiatric Association, 2013). Stuttering that persists into adulthood can lead to significant restrictions in quality of life and social and professional development (McAllister et al., 2012).

The most frequent form of stuttering, childhood onset speech fluency disorder, occurs in at least 5% of all children and typically presents between the ages of 3 and 6 years (Bloodstein and Ratner, 2008; Reilly et al., 2013). Recovery frequently occurs within the first years, particularly in girls. Stuttering persists after puberty in approximately 1% of the general population, with a male to female ratio of 4 to 1 (Yairi and Ambrose, 2013). Currently, access to treatment is limited by regional availability (Donaghy and Smith, 2016).

Since 1979, the German Stuttering Association has aimed to “counteract the development of stuttering and to improve the living situation of people who stutter” (Bundesvereinigung Stottern & Selbsthilfe e.V., 2020), an effort that has included performing critical reviews of available therapies in terms of accessibility, evidence, and efficiency.

In dialogue with therapists, and during the creation of the guidelines for speech fluency disorders (Neumann et al., 2017), the scarcity of data regarding the current state of stuttering therapy in Germany was emphasized (Radtke, 2019; Waltersbacher, 2019). Questions regarding (1) the frequency with which stuttering is diagnosed in Germany and (2) the form, intensity, and duration of current stuttering therapies in Germany were raised. Another unanswered question was (3) whether the intensity of speech therapy (intensive therapy lasting several weeks compared with one or two weekly sessions for several months) had any relevant impacts on the success or the duration of therapy.

Answering these questions will provide insight into current treatment realities, will help identify treatment traditions and patterns, and might encourage a debate on optimized use of treatment resources for stuttering, cluttering, and developmental disorders of speech in Germany and other countries.

To understand how timely the diagnosis is made, and how timely treatment is initiated, we also assessed the proportion of newly diagnosed patients in the year 2017, and the proportion of treatment allocation in the first year of diagnosis.

Co-existing disorders may influence the long-term response to treatment (Iverach et al., 2009) and might shed light on potential underlying disease mechanisms. A range of disorders has been reported to occur more frequently among individuals who stutter. We used this large database to verify or refute these reports, and contrasted it with the comorbidities of cluttering as well as developmental disorders of speech and language.

We also assessed data on developmental disorders of speech and language, as well as on cluttering. Speech and language abilities are key factors for successful schooling and career development (McAllister et al., 2012; Dubois et al., 2020), and they have received increasing attention in Germany after the relatively poor performance of German pupils during the early runs of the Program for International Student Assessment (PISA)^1^ (Turner and Adams, 2007). Cluttering, on the other hand, is a rare disorder characterized by a speech rate that is perceived to be abnormally rapid, with some overlap with stuttering (Myers et al., 2012; Bona, 2019). Specific developmental disorders refer to disorders in which development is delayed in one specific area, such as speech and language, which can present with a broad range of clinical characteristics (Neumann et al., 2009) without affecting other areas of development. These disorders provide a useful background and context for the data on stuttering.

## Materials and Methods

This study was performed as a retrospective analysis of secondary data, conducted using the research database of Wissenschaftliches Institut der AOK (Allgemeine Ortskrankenkassen) (WIdO, Research Institute of the Local Health Care Funds, Berlin, Germany). AOK is the largest sickness fund group within Germany’s statutory health insurance system, able to provide access to the medical details of approximately 32% of the total German population (Nimptsch et al., 2014; Busse et al., 2017; Karagiannidis et al., 2020). 87.7% of citizens in Germany have statutory health insurance (Federal Ministry of Health, 2020), and membership is open to everyone, regardless of factors such as profession, income, age, or comorbidities (Busse et al., 2017; Karagiannidis et al., 2020). Available data were anonymous at the patient level but included patient characteristics, such as age, sex, diagnosis, admissions as inpatients, practitioner consultations, medications used, and other items associated with the use of healthcare services. In Germany, physicians’ claims must be submitted at the end of each quarter, generating four time units for each year in the dataset, with each unit representing a 3-month period. In total, 16 quarters were available for the consecutive insurance years of 2015–2018. The study was approved by the ethics committee of the University of Frankfurt. No funding sources were obtained for this study. STROSA guidelines (Standardized Reporting Of Secondary data Analyses) were followed (Swart et al., 2016).

### Identification of the Study Population (Annual Prevalence)

Medical records that included the codes for stuttering (F98.5), cluttering (F98.6), and developmental disorders of speech and language (F80), based on the ICD-10-GM (10th revision of the International Statistical Classification of Diseases and Related Health Problems, German Modification)^2^, were used to identify patients with disorders of speech and language. At the level of the third and fourth digits, the codes used for the ICD-10 and ICD-10-GM are not discernibly different; therefore, this article will refer to the ICD-10. The ICD-10 coding has previously been used in Germany and other countries to identify cases of brain disorders, demonstrating sensitivity and positive predictive values of up to 98% (Reid et al., 2012; St Germaine-Smith et al., 2012; Jette et al., 2015; Ertl et al., 2016; Strzelczyk et al., 2017a, b, 2021; Schubert-Bast et al., 2019). To ensure the classification validity of speech and language disorders, patients included in this analysis were required to meet the requirements of an ensured diagnosis, which included at least one confirmed outpatient diagnosis of F98.5, F98.6, or F80 during at least one quarter of the insurance year of interest. As the German healthcare system offers eleven regular preventive screening examinations to all children [“Vorsorgeuntersuchung” U1 (at birth) – U11 (age 9–10 years)] and two examinations to adolescents at age 12–14 years (J1) and 16/17 years (J2), we assume a rigorous ICD-10 coding. Presentation to the preventive screening examinations is mandatory in some German states or rigorously controlled. We included all ages in these analyses. After assessing the entire sample, we took a detailed look on the subgroups diagnosed with stuttering (F98.5) or cluttering (F98.6).

### Identification of Newly Diagnosed Patients (Incidence Population)

To analyze the time at which a disorder of speech and language was diagnosed, newly diagnosed patients within the insurance reporting system were identified. A newly diagnosed disorder of speech and language was assumed for those patients with no ensured diagnosis of any speech and language disorder during the previous 2 years of observation (i.e., 2015 and 2016), and two confirmed diagnoses of F98.5, F98.6, or F80 that were coded during 2017 or during the first quarter of 2018. Thus, the annual incidence was provided for patients older than 3 years starting in the year 2017. Again, we took a detailed look on the subgroups diagnosed with stuttering (F98.5) or cluttering (F98.6) after assessing the entire sample.

### Treatment Calculations

Speech therapy is prescribed by physicians, and the costs are covered by the statutory health insurance, coded as X3001--X3224 (Bundeseinheitliches Heilmittelpositionsnummernverzeichnis)^3^. The proportion of patients who were treated with speech therapy and the frequency of the treatment sessions (typically 45 min in length) were calculated for the above-defined populations, irrespective of the diagnosis provided on the prescription for speech therapy (Waltersbacher, 2014).

### Comorbidities

As the diagnosis of stuttering could also be related to acquired stuttering, we analyzed in detail the occurrence of comorbidities in the cohorts with speech disorders and the total insured population. For the insurance year 2017, we evaluated the co-occurrence of disorders and comorbidities for which a co-existence with stuttering has previously been reported in the literature, including: anxiety disorders (ICD10 codes F40.x, F41.x, and F93.0) (Iverach et al., 2009); ADHD (F90.x) (Ajdacic-Gross et al., 2018); tic disorders and Tourette syndrome (F95.x) (Ajdacic-Gross et al., 2018); personality disorders (F60.x, F61.x, and F62.x) (Iverach et al., 2009); specific developmental disorders of scholastic skills (F81.x) (Ajdacic-Gross et al., 2018); atopic disorders (J30.1–J30.4, J45.0, L20.x, and J30.1–J30.4) (Ajdacic-Gross et al., 2020); mental retardation (F70–F74); chromosomal anomalies (Q90x–Q99.x); and neurodevelopmental disorders (G40.x, G80.x. G91.x, G93.0, and G93.1) (Neumann et al., 2017).

### Statistical Analysis

All data were managed and analyzed using an anonymous patient code to comply with data protection regulations. Data were analyzed using Db Visualizer Pro 10.0.13/Toad Data Point/Excel. The annual crude (i.e., non-adjusted against the total population) prevalence rates were calculated based on the number of cases identified in the study years 2015–2018, divided by the total number of AOK-insurants per year. Because the study was intended to be explorative in nature, no further adjustments for multiple testing were performed. To evaluate the incidence of comorbidities, we calculated the percentages of affected individuals among the total number of individuals affected with F80, F98.5, or F96.5 and compared these values with the percentage of affected individuals among the general population of AOK-insurants aged 0–19 years by calculating odds ratios.

## Results

### Identification of the Entire Study Population

We identified 585,551 patients (insurance year 2017, crude prevalence of 2.13% among the total AOK-insured population of 27.5 million people) who met our definition for diagnosis with a disorder of speech and language. Among these patients, 358,294 were male (61.2%, crude prevalence of 2.63%) and 227,257 were female (38.8%, crude prevalence of 1.63%). Table 1 shows the study population and the crude prevalence rates for the years 2015–2018. Although the crude prevalence increased from 2.00% to 2.24% during this period, the ratio between males and females remained constant, at 1.57 to 1.

**TABLE 1:** Annual total number of patients and crude annual prevalence of disorders of speech and language coded with at least one confirmed ICD-10 diagnosis of developmental disorders of speech and language, stuttering, or cluttering.

Insurance year	Male patients *n* (%)	Female patients *n* (%)	Total number of patients	Crude annual prevalence among males	Crude annual prevalence among females
2015	315,959 (61.2)	200,265 (38.8)	516,224	2.51%	1.51%
2016	334,047 (61.2)	212,175 (38.8)	546,222	2.53%	1.56%
2017	358,294 (61.2)	227,257 (38.8)	585,551	2.63%	1.63%
2018	383,924 (61.1)	244,072 (38.9)	627,996	2.76%	1.73%

**FIGURE 1:**
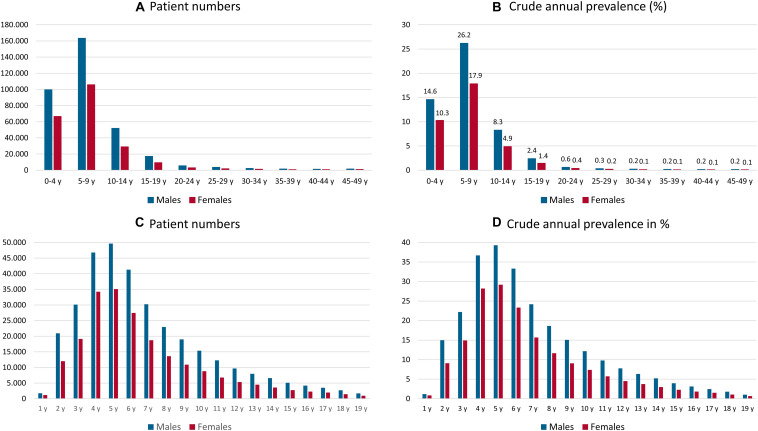
Annual total number **(A,C)** and crude annual prevalence **(B,D)** of male and female patients in each 5-year age group **(A,B)** and each 1-year age group [1–19 years; **(C,D)**] with of a disorder of speech and language coded with a diagnosis of developmental disorders of speech and language, stuttering, or cluttering for insurance year 2017; y = years.

Disorders of speech and language show a characteristic peak between the ages of 5 and 9 years, with a crude prevalence of 22.1% and predominance among males (crude prevalence of 26.2%) compared with females (17.9%). Disorders of speech and language are rarely coded in patients older than 20 years of age. A detailed analysis of the prevalent cases and crude incidence rates for each age group by year until the age of 19 years showed a peak at the age of 5 years (crude prevalence of 39.3% in males and 29.2% in females). Details regarding the age and gender distributions of disorders of speech and language are shown in Figure 1.

### Identification of Prevalent Patients With Stuttering and Cluttering

**FIGURE 2:**
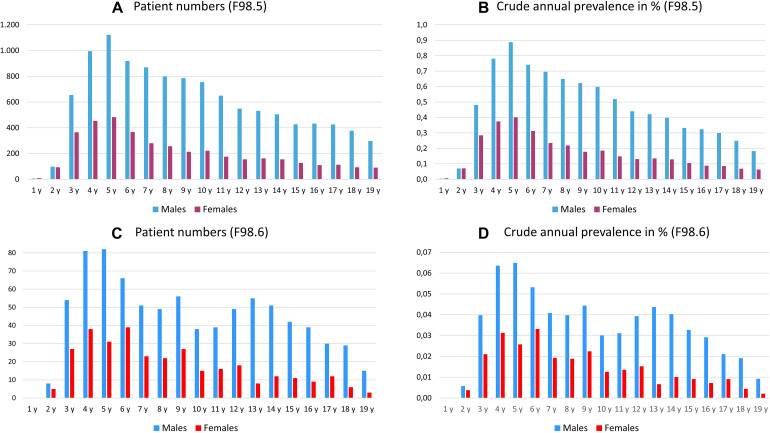
Annual total number **(A,C)** and crude annual prevalence [in%, **(B,D)**] of male and female patients in each 1-year age group diagnosed with stuttering **(A,B)**, stuttering or cluttering **(C,D)**, in insurance year 2017; y = years.

In the entire dataset, we identified 27,977 (insurance year 2017) patients of all age groups who were diagnosed with stuttering, including 21,045 males (75%) and 6,932 females (25%). The overall crude prevalence across age and gender was 0.102%. The prevalence of stuttering shows a characteristic peak at the age of 5 years, with a crude annual prevalence of 0.65% (annual prevalence of 5-year-old patients) and predominance in males (crude prevalence of 0.89%) as compared with females (0.40%). The distributions by age and sex are shown in Figures 2A,B. During the analyzed insurance years of 2015–2018, the ratios between males and females remained constant at 2.78–2.86 to 1.

Cluttering was diagnosed in 1,800 (insurance year 2017) patients among all age groups, including 1,287 males (71.5%) and 513 females (28.5%). The overall prevalence across age and gender was 0.0013%. The prevalence of cluttering peaked in the age group from 4 to 6 years, with a crude prevalence of between 0.043 and 0.048% and predominance in males (crude prevalence of 0.053–0.065%) compared with females (0.026–0.033%). The distributions by age and sex are shown in Figures 2C,D. These numbers appeared too small to warrant inclusion into the subsequent analyses.

### Identification of Annual Newly Diagnosed Cases

**FIGURE 3:**
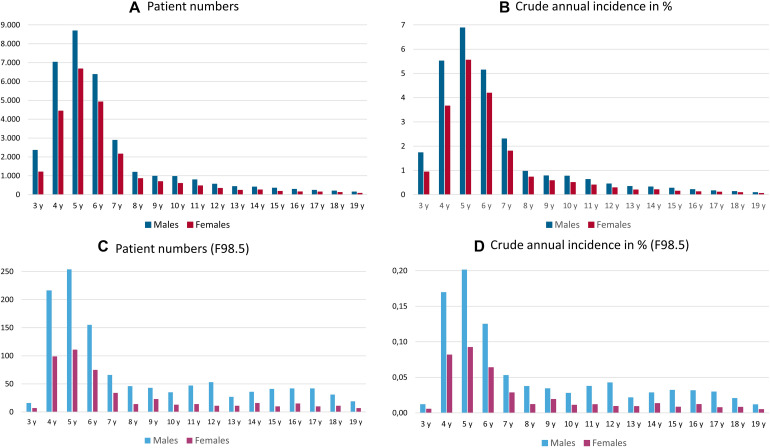
Annual total number **(A,C)** and the crude annual incidence [in%, **(B,D)**] of male and female patients in each 1-year age group diagnosed with a disorder of speech and language **(A,B)**, as coded by at least one confirmed ICD-10 diagnosis of developmental disorders of speech and language, stuttering, or cluttering, and those diagnosed with stuttering **(C,D)** in insurance year 2017; y = years.

The number of newly diagnosed patients who were coded with a disorder of speech and language was calculated for the insurance year 2017, and the details are presented in Figure 3, from the age of 3 years through the age of 19 years. Incident patients peaked among the ages of 4–6 years before declining steadily with increasing age.

### Use of Speech Therapy in Newly Diagnosed Cases and Prevalent Population

**FIGURE 4:**
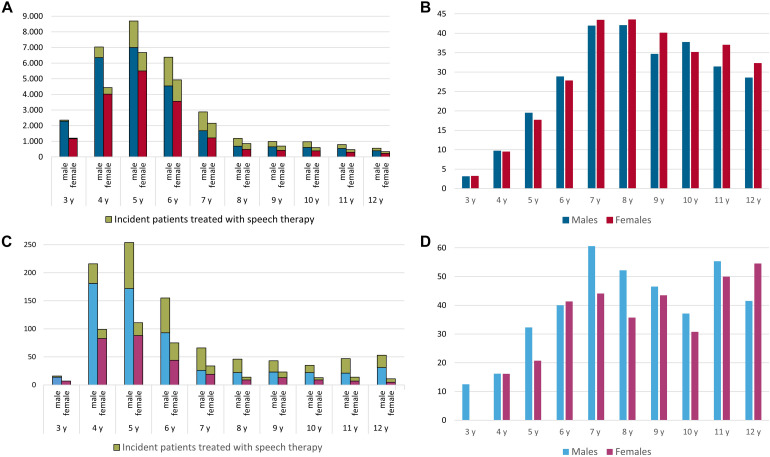
Use of speech therapy in newly diagnosed patients (green color: speech therapy, blue colors: males, red colors: females) with a disorder of speech and language **(A,B)**, as coded by at least one confirmed ICD-10 diagnosis of developmental disorders of speech and language, stuttering, or cluttering, and those diagnosed with stuttering **(C,D)** for insurance year 2017; y = years.

The onset of speech therapy in the same year as a disorder of speech and language was newly diagnosed peaked at the ages of 7 and 8 years (42.5 to 42.6%). The details are provided in Figures 4A,B. The onset of speech therapy among children diagnosed with stuttering was earlier and represented a higher proportion of newly diagnosed patients, including 40.4% at 6 years, 55% at 7 years, 48.3% at 8 years, and 45.4% at 9 years. The details are presented in Figures 4C,D. During the year of incident diagnosis, a mean of 15.5 therapy sessions were prescribed for disorders of speech and language, compared with a mean of 13.5 therapy sessions for children with stuttering.

**FIGURE 5:**
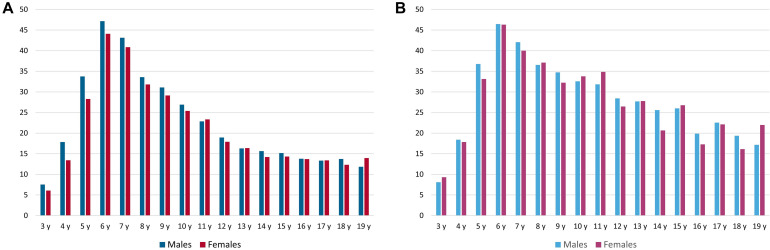
The percentage of prevalent patients in each 1-year age group prescribed speech therapy (in%) to treat a disorder of speech and language **(A)**, coded with at least one confirmed ICD-10 diagnosis of developmental disorders of speech and language, stuttering, or cluttering), and to treat stuttering **(B)** in insurance year 2017; y = years.

The percentage of prevalent cases of a disorder of speech and language who were prescribed speech therapy peaked at the ages of 6 (45.9%) and 7 (42.2%) years and decreased to below 20% starting at the age of 12 years. The details are provided in Figure 5A. Each year, a mean of 21.2 therapy sessions were prescribed for patients with disorders of speech.

In children with stuttering, the percentage of prevalent cases who were prescribed speech therapy also peaked at the ages of 6 (46.4%) and 7 (41.6%) years and decreased below 20% starting at the age of 16 years. The details are presented in Figure 5B. Each year, a mean of 19.8 therapy sessions were prescribed for patients diagnosed with stuttering. The details of speech therapy sessions, according to gender and age group, are provided in Table 2.

**TABLE 2:** Annual total number and percentage of treated prevalent patients and the frequency of speech therapy among males and females in each 1-year age group diagnosed with stuttering in insurance year 2017; y = years.

Age groups	Number of males	Number of females	% of males with speech therapy	% of females with speech therapy	Mean number of speech therapy sessions in males	Mean number of speech therapy sessions in females
2 y	98	93	2.0	1.1	6.0	7.0
3 y	654	365	8.1	9.3	10.7	10.9
4 y	995	454	18.4	17.8	17.4	16.2
5 y	1,121	483	36.8	33.1	21.8	20.7
6 y	919	367	46.5	46.3	21.8	21.9
7 y	870	280	42.1	40.0	20.2	21.4
8 y	799	256	36.5	37.1	18.7	19.3
9 y	786	214	34.7	32.2	20.5	20.3
10 y	755	222	32.6	33.8	19.5	19.0
11 y	650	175	31.8	34.9	19.1	17.3
12 y	549	155	28.4	26.5	19.2	20.2
13 y	531	162	27.7	27.8	21.4	16.2
14 y	504	155	25.6	20.6	19.2	22.6
15 y	427	127	26.0	26.8	20.9	19.7
16 y	433	110	19.9	17.3	17.4	15.1
17 y	426	113	22.5	22.1	18.6	17.4
18 y	377	93	19.4	16.1	18.4	18.5
19 y	297	91	17.2	22.0	17.9	19.3
***Total population aged 2–19 y***	***Total11,191***	***Total3,915***	***Mean29.6***	***Mean27.7***	***Mean19.9***	***Mean19.5***

### Concomitant Disorders and Comorbidities

Based on the comorbidities that have been reported in the literature, the prevalences of concomitant disorders and comorbidities were examined among patients coded with at least one confirmed ICD-10 diagnosis of developmental disorders of speech and language (F80), stuttering (F98.5), or cluttering (F98.6). These numbers were compared against the prevalences observed among the general insurance population aged 0–19 years for the insurance year 2017. Table 3 shows elevated odds ratios in patients with developmental disorders of speech and language (F80) compared with those for the general population for: atopic dermatitis (L20) (Ajdacic-Gross et al., 2020); phobic disorders (F40, F41, and F93) (Iverach et al., 2009); ADHD (F90) (Ajdacic-Gross et al., 2018); tic disorders and Tourette’s syndrome (F95) (Ajdacic-Gross et al., 2018); personality disorders (F60) (Iverach et al., 2009); specific developmental disorders of scholastic skills (F81) (Ajdacic-Gross et al., 2018); intellectual disabilities (F70–F74); chromosomal anomalies (Q90 and Q93); and neurodevelopmental disorders (G40, G80, G91, G93, and G93) (Neumann et al., 2017). Table 4 shows a similar distribution of concomitant disorders and comorbidities among patients with stuttering (F98.5), whereas Table 5 shows the distribution among those diagnosed with cluttering (F98.6).

**TABLE 3:** Prevalences of concomitant disorders and comorbidities among the total insured population, aged 0–19 years, and among those with at least one confirmed ICD-10 diagnosis of developmental disorders of speech and language (F80).

ICD-10 code	Disorders/Comorbidities	Male (Total 2,649,451)	%	Male (F80 323,077)	%	Odds ratio	Female (Total 2,492,211)	%	Female (F80 207,086)	%	Odds ratio
L20	Atopic dermatitis	218,016	8.23	40,556	12.55	1.74	214,572	8.61	26,348	12.72	1.76
F90	Attention-deficit hyperactivity disorders	139,446	5.26	34,649	10.72	2.55	48,850	1.96	11,190	5.40	3.71
F91	Conduct disorders	63,902	2.41	21,217	6.57	3.76	33,305	1.34	8,514	4.11	4.25
F81	Specific developmental disorders of scholastic skills	58,529	2.21	17,936	5.55	3.31	35,816	1.44	9,407	4.54	4.43
F41	Other anxiety disorders	22,232	0.84	3,522	1.09	1.36	34,959	1.40	2,711	1.31	1.01
F92	Mixed disorders of conduct and emotions	21,818	0.82	6,052	1.87	2.80	12,882	0.52	2,501	1.21	2.92
G40	Epilepsy	20,285	0.77	5,374	1.66	2.62	16,958	0.68	3,506	1.69	3.17
F95	Tic disorders	15,672	0.59	3,752	1.16	2.28	6,684	0.27	1,159	0.56	2.53
F40	Phobic anxiety disorders	12,463	0.47	2,489	0.77	1.80	16,671	0.67	1,757	0.85	1.42
G80	Cerebral palsy	8,455	0.32	2,696	0.83	3.39	6,196	0.25	1,715	0.83	4.63
F70	Mild intellectual disabilities	8,281	0.31	3,910	1.21	6.51	4,944	0.20	1,999	0.97	8.23
F60	Specific personality disorders	6,671	0.25	1,593	0.49	2.27	11,109	0.45	1,085	0.52	1.30
Q90	Down syndrome	3,172	0.12	1,569	0.49	7.08	2,569	0.10	1,254	0.61	11.53
G91	Hydrocephalus	3,080	0.12	823	0.25	2.63	2,076	0.08	457	0.22	3.40
F71	Moderate intellectual disabilities	2,836	0.11	1,333	0.41	6.41	1,616	0.06	715	0.35	9.58
Q99	Other chromosome abnormalities not elsewhere classified	2,351	0.09	1,234	0.38	7.98	1,701	0.07	725	0.35	8.96
F72	Severe intellectual disabilities	1,486	0.06	634	0.20	5.37	971	0.04	377	0.18	7.65
Q93	Monosomies and deletions from the autosomes. not elsewhere classified	714	0.03	356	0.11	7.17	724	0.03	319	0.15	9.49

*The numbers and percentages of diagnosed patients and odds ratios are provided for male and female AOK-insurants for insurance year 2017.*

**TABLE 4:** Prevalences of concomitant disorders and comorbidities among the total insured population, aged 0–19 years, and for those with at least one confirmed ICD-10 diagnosis of stuttering (F98.5).

ICD-10 code	Disorders/Comorbidities	Male (Total 2,649,451)	%	Male (F98.5 11,096)	%	Odds ratio	Female (Total 2,492,211)	%	Female (F98.5 3,896)	%	Odds ratio
L20	Atopic dermatitis	218,016	8.23	1,215	10.95	1.37	214,572	8.61	488	12.53	1.52
F90	Attention-deficit hyperactivity disorders	139,446	5.26	1,299	11.71	2.40	48,850	1.96	186	4.77	2.51
F91	Conduct disorders	63,902	2.41	683	6.16	2.67	33,305	1.34	152	3.90	3.00
F81	Specific developmental disorders of scholastic skills	58,529	2.21	673	6.07	2.88	35,816	1.44	176	4.52	3.24
F41	Other anxiety disorders	22,232	0.84	227	2.05	2.48	34,959	1.40	105	2.70	1.95
F92	Mixed disorders of conduct and emotions	21,818	0.82	213	1.92	2.37	12,882	0.52	52	1.33	2.60
G40	Epilepsy	20,285	0.77	175	1.58	2.09	16,958	0.68	48	1.23	1.82
F95	Tic disorders	15,672	0.59	289	2.60	4.56	6,684	0.27	60	1.54	5.82
F40	Phobic anxiety disorders	12,463	0.47	105	0.95	2.03	16,671	0.67	55	1.41	2.13
G80	Cerebral palsy	8,455	0.32	51	0.46	1.44	6,196	0.25	26	0.67	2.70
F70	Mild intellectual disabilities	8,281	0.31	145	1.31	4.28	4,944	0.20	30	0.77	3.90
F60	Specific personality disorders	6,671	0.25	81	0.73	2.94	11,109	0.45	37	0.95	2.14
Q90	Down syndrome	3,172	0.12	19	0.17	1.43	2,569	0.10	12	0.31	2.99
G91	Hydrocephalus	3,080	0.12	18	0.16	1.40	2,076	0.08	2	0.05	–*
F71	Moderate intellectual disabilities	2,836	0.11	43	0.39	3.67	1,616	0.06	15	0.39	5.96
Q99	Other chromosome abnormalities not elsewhere classified	2,351	0.09	28	0.25	2.87	1,701	0.07	2	0.05	–*
F72	Severe intellectual disabilities	1,486	0.06	10	0.09	1.61	971	0.04	2	0.05	–*

The numbers and percentages of diagnosed patients and odds ratios are provided for male and female AOK-insurants in insurance year 2017. *Odds ratio not provided due to the limited number of affected individuals.

**TABLE 5:** Prevalences of concomitant disorders and comorbidities among the total insured population, aged 0–19 years, and for those with at least one confirmed ICD-10 diagnosis of cluttering (F98.6).

ICD-10 code	Disorders/Comorbidities	Male (Total 2,649,451)	%	Male (F98.6 829)	%	Odds ratio	Female (Total 2,492,211)	%	Female (F98.6 319)	%	Odds ratio
L20	Atopic dermatitis	218,016	8.23	93	11.22	1.41	214,572	8.61	45	14.11	1.74
F90	Attention-deficit hyperactivity disorders	139,446	5.26	160	19.30	4.31	48,850	1.96	20	6.27	3.35
F91	Conduct disorders	63,902	2.41	58	7.00	3.05	33,305	1.34	19	5.96	4.68
F81	Specific developmental disorders of scholastic skills	58,529	2.21	76	9.17	4.47	35,816	1.44	21	6.58	4.83
F41	Other anxiety disorders	22,232	0.84	13	1.57	1.88	34,959	1.40	6	1.88	–*
F92	Mixed disorders of conduct and emotions	21,818	0.82	21	2.53	3.13	12,882	0.52	9	2.82	–*
G40	Epilepsy	20,285	0.77	10	1.21	1.58	16,958	0.68	2	0.63	–*
F95	Tic disorders	15,672	0.59	17	2.05	3.52	6,684	0.27	4	1.25	–*
F40	Phobic anxiety disorders	12,463	0.47	9	–*	2.32	16,671	0.67	6	1.88	–*
G80	Cerebral palsy	8,455	0.32	4	–*	1.51	6,196	0.25	2	0.63	–*
F70	Mild intellectual disabilities	8,281	0.31	14	1.69	5.49	4,944	0.20	3	0.94	–*
F60	Specific personality disorders	6,671	0.25	8	–*	3.86	11,109	0.45	7	2.19	–*

The numbers and percentages of diagnosed patients and odds ratios are provided for male and female AOK-insurants for insurance year 2017. *Odds ratio not provided due to the limited number of affected individuals.

Furthermore, were analyzed the ICD-10 coding overlap between children and adolescents with stuttering, cluttering and disorders of speech and language. Among those diagnosed with stuttering the overall crude prevalence of cluttering was 1.2% (1.3% in males and 0.9% in females), and 48.3% for disorders of speech and language (48.9% in males and 46.8% in females). Among children and adolescents diagnosed with cluttering the overall crude prevalence of stuttering was 15.7% (17.3% in males and 11.6% in females), and 57.3% for disorders of speech and language (58.5% in males and 54.2% in females). The overall crude prevalence of stuttering was 1.37% (1.68% in males and 0.88% in females), and 0.12% for cluttering (0.15% in males and 0.08% in females) in those diagnosed with disorders of speech and language.

## Discussion

This study represents the first nationwide study to use German health insurance data to analyze the incident and prevalent diagnoses of a disorder of speech and language, including stuttering (F98.5), cluttering (F98.6), and developmental disorders of speech and language (F80), and to evaluate the speech therapy treatment patterns.

Recently, an increasing proportion of children have received treatment for language development disturbances, which has been the focus of lively debate. Our data are consistent with this reported nationwide trend. Table 1 shows that an increasing percentage of the total insured population has been diagnosed with an ICD-10 diagnosis of developmental disorders of speech and language, stuttering, or cluttering. This increase can largely be attributed to developmental disorders of speech and language, whereas the proportion of stuttering individuals among insurance members has remained relatively stable, at approximately 0.3% of the total population (Supplementary Table 1).

The age distribution observed in this study is consistent with the known epidemiology of speech and language disorders, which peak at approximately 4 to 5 years of age, which is a phase of active language development. For stuttering, this peak is earlier, at 30–36 months of age (Mansson, 2000; Bloodstein and Ratner, 2008). Our conservative condition of two preceding years without the diagnosis might have shifted incidence peaks slightly toward older ages.

The annual crude prevalence of stuttering peaked in our sample among patients 5 years of age, which represented 0.65% of the total population. This finding is consistent with the literature summarized in Chapter 3 of Bloodstein and Ratner (2008). In contrast with annual prevalence, as analyzed in this study, the frequent statement that “five percent of all children stutter” (Walsh et al., 2015) refers to the cumulative lifetime prevalence of stuttering. However, we did not analyze longitudinal data; therefore, we cannot provide a lifetime prevalence for our cohort.

In our dataset, the gender ratio for all speech and language disorders favors girls, who are less affected, and remained stable across all the years included in the database under study. This is consistent with the literature (McLeod and McKinnon, 2007; Arrhenius et al., 2018). With regard to stuttering, a yet unresolved question is whether the gender imbalance increases over the years, which would be expected because spontaneous recovery is observed more frequently in girls than in boys (Yairi and Ambrose, 2013; Kefalianos et al., 2017); however, this assumption is not universally supported by population-based data (National Institute on Deafness and Other Communication Disorders, 2010). Unfortunately, such longitudinal insights cannot be derived from our cross-sectional dataset.

The allocation of treatment resources is another debated topic (Wissenschaftliches Institut der AOK, 2012). Our population-based study yielded novel insights regarding treatment prescription behavior. Some affected individuals did not receive therapy, although the diagnosis-based coding system used by ICD-10 does not reflect disease severity or the need for therapy. Therefore, a substantial proportion of affected individuals may not seek therapy because they are only mildly or briefly affected. Indeed, spontaneous recovery occurs early, typically within the first months of speech dysfluency, and most patients recover within the first 2 years after disease onset (Lattermann, 2011), although the extent of early recovery is debated (Reilly et al., 2013).

In our sample, approximately 45% of individuals diagnosed with stuttering received dedicated speech therapy within the year of receiving a coded ICD-10 diagnosis. In Germany, mandatory child screening examinations performed by pediatricians are associated with a high attendance rate, rendering the possibility of underdiagnosis unlikely (Schmidtke et al., 2018; Santos et al., 2020), at least for moderate or severe cases (Winters and Byrd, 2020). In addition, the diagnostic tools available to evaluate preschoolers have improved (Neumann et al., 2014a, b). In our sample, a striking peak in treatment allocation was observed starting at 6 years of age, which coincides with the beginning of regular schooling in Germany, a milestone that may trigger increased demand for treatment. This is difficult to reconcile with the current recommendations for a maximum wait-and-see delay of 1 year (Neumann et al., 2017). Because earlier therapy is likely more able to induce lasting recovery (Jones et al., 2005; Neumann et al., 2017), the current peak of therapy at the time of school entrance may indicate the possibility of earlier treatment. In Germany, speech language treatment usually does not take place in schools. Hence, frequency and spacing of therapy are not necessarily determined by traditions of school scheduling.

The prevalence of treatment in our study was higher with 45% during the first year of diagnosis than in the Australian Early Language in Victoria Study (Kefalianos et al., 2017). Of those parents who reported about seeking treatment by the age of 7 years, only 16.7% of children with persistent stuttering had received intervention for stuttering at some point during the preschool years. In those who had recovered from stuttering only 13.4% received stuttering treatment. At the age of 7 years, 39% of parents of children with persistent stuttering reported that they had sought help or advice for their child’s stuttering at some point during the preschool years, whereas 28% of parents of children who had recovered from stuttering reported seeking help or advice (Kefalianos et al., 2017).

The yearly mean number of therapy sessions for stuttering was slightly lower than 20, reflecting the receipt of two of the usual prescriptions for ten treatment sessions. Treatment frequency is usually once per week. We cannot infer the total treatment duration because we analyzed yearly, partially independent, cross-sectional samples rather than longitudinal data. Therefore, total treatment duration or intensification of therapy over time cannot be inferred. By comparison, the mean number of treatments for developmental disorders of speech and language was comparable, at 21 sessions per year. Of note, there is no formal upper limit of sessions per case per year. In practice, the limit is the number of prescriptions for a single case issued and signed by the pediatricians.

Although the neurological background of stuttering is increasingly understood (Neef et al., 2015), the factors that influence the evolution toward recovery or persistency remain elusive. Comorbidities, such as anxiety or other psychiatric disorders, increase the likelihood of relapse following treatment (Iverach et al., 2009; Menzies et al., 2014). A higher prevalence of comorbidities among the cohorts with stuttering, cluttering, or developmental disorders of speech and language could be confirmed in this study. In addition, atopic disorders, such as hay fever, have been shown to be associated with stuttering persistency (Strom and Silverberg, 2016; Ajdacic-Gross et al., 2020). In our sample, we could substantiate this evolving matter by showing increased odds ratios for atopic disorders. A putative interaction between the two groups of disorders remains to be elucidated and is beyond the scope of this explorative study.

### Limitations

With the data at hand, we were unable to address the other questions raised in the introduction. In particular, intensive inpatient therapy settings in Germany are prescribed outside of the ordinary therapy prescription and reimbursement procedures and are not covered by the present dataset.

A distortion might arise from the fact that the apparent coding does not permit identifying primary and secondary disorders. For example, if a child has hydrocephalus or chromosomal defects that are severe, it is likely that stuttering or cluttering may well never be considered as a salient disorder.

Pure cluttering was only rarely coded, even in this large database, with numbers that were too low for further analyses. Analyzing this disorder may require dedicated patient sampling from a nationwide sample.

Online speech therapy has been pioneered in Australia (O’Brian et al., 2008) and is increasingly being used (Wolff Von Gudenberg and Euler, 2017) recently due to the limits on face-to-face therapy that have been imposed in response to the coronavirus disease 2019 (COVID-19) pandemic. Whether this change of setting will affect the efficacy and outcome of treatment has not yet been determined.

Even if the reported results are based on a large database, no statement can be made about all residents in Germany. The nationwide population-based studies, for example by the Robert Koch Institute on adult health in Germany, also show clear differences between the various types of statutory health insurance (Hoffmann and Icks, 2012; Hoffmann and Koller, 2017). An extrapolation procedure developed by the WIdO together with the Chair of Economic and Social Statistics at the University of Trier, which takes into account different age and gender structures as well as additional morbidity differences, can currently be used to estimate the prevalence of all residents of Germany (Breitkreuz et al., 2019). A corresponding extrapolation method that also compensates for the differences in health care between the populations has not yet been developed. However, we assume a good coding of any neurodevelopmental or speech disorders in childhood and adolescence as the German healthcare system offers thirteen regular preventive screening examinations from birth to the age of 17 years that are mandatory is some German states or rigorously controlled in other. Therefore it seems unlikely that speech disorders might be not recognized at all during childhood and adolescence (Santos et al., 2020).

## Conclusion

The significance of this study arises from the analysis of health insurance data for a sample population that represents 32% of the German population. This study represents the first time that such an analysis has been performed for a disorder of speech and language. Prevalence and gender ratio data were consistent with the international literature. The crude prevalence of developmental disorders of speech and language increased from 2015 to 2018, whereas the crude prevalence for stuttering remained stable. For cluttering, the numbers were too low to draw reliable conclusions. Proportional treatment allocation for stuttering peaked at 6 years of age, which is the school entrance year, and is later than the prevalence peak of stuttering. Future analyses should explore whether new approaches to treatment could improve outcomes for severely affected patients. Follow-up longitudinal studies will allow an even better characterization of treatment intensity and duration.

## Data Availability Statement

The original contributions presented in the study are included in the article/Supplementary Material, further inquiries can be directed to the corresponding author/s.

## Author Contributions

MS and ASt developed the idea for this study and conceived the manuscript. AW collected the data. MS, AW, and ASt performed the statistical analysis. ASt created the tables and figures. All authors wrote the manuscript, discussed the results, contributed to the final manuscript, and approved the final manuscript for publication.

## Conflict of Interest

MS serves as chairman of the German Stuttering Association. He reports personal fees and grants from Deutsche Forschungsgemeinschaft, Primate Cognition (Leibniz-WissenschaftsCampus), Scientific Organizations (EFCN, UCL, DGKN, and IVS), and pharmaceutical companies (Novartis, GlaxoSmithKline, UCB, and Medtronic), all outside the submitted work. AW, Projektleiterin AOK-Heilmittel-Informations-System; HS, Stellvertretender Geschäftsführer WIdO; and ASc, research associate, are employees of Wissenschaftliches Institut der AOK. ASt reports personal fees and grants from Arvelle Therapeutics, Desitin Arzneimittel, Eisai, GW Pharmaceuticals, LivaNova, Marinus Pharmaceuticals, Medtronic, UCB Pharma, and Zogenix, all outside the submitted work.

## Footnotes

1 www.oecd.org/berlin/themen/pisa-studie/

2 www.dimdi.de

3 https://www.gkv-datenaustausch.de/leistungserbringer/sonstige_leistungserbringer/positionsnummernverzeichnisse/positionsnummernverzeichnisse.jsp

## Supplementary Material

Supplementary Table 1 Annual total number of AOK-insurants below 20 years of age and crude annual prevalence (left side) with at least one confirmed ICD-10 diagnosis of developmental disorders of speech and language (F80), stuttering (F98.5) or cluttering (F98.6); or (right side) with at least one confirmed ICD-10 diagnosis of stuttering (F98.5).
