# Supplementary material for: Prevalence and Therapy Rates for Stuttering, Cluttering, and Developmental Disorders of Speech and Language: Evaluation of German Health Insurance Data
Source: Front Hum Neurosci. 2021 Apr 12;15:645292. doi: 10.3389/fnhum.2021.645292 (PMC8071871; doi:10.3389/fnhum.2021.645292)
Supplement: Supplementary Table 1 — Annual total number of AOK-insurants below 20 years of age and crude annual prevalence (left side) with at least one confirmed ICD-10 diagnosis of developmental disorders of speech and language (F80), stuttering (F98.5) or cluttering (F98.6); or (right side) with at least one confirmed ICD-10 diagnosis of stuttering (F98.5). [file Table_1.docx]

Supplementary material:

Supplementary Table 1: *Annual total number of AOK-insurants below 20 years of age and crude annual prevalence (left side) with at least one confirmed ICD-10 diagnosis of developmental disorders of speech and language (F80), stuttering (F98.5) or cluttering (F98.6); or (right side) with at least one confirmed ICD-10 diagnosis of stuttering (F98.5).*
